# Supplementary material for: Are carbon emissions trading and green financial instruments synergistic? -Comprehensive quantitative research based on content analysis
Source: PLoS One. 2024 Mar 7;19(3):e0298601. doi: 10.1371/journal.pone.0298601 (PMC10919601; doi:10.1371/journal.pone.0298601)
Supplement: S1 Data — (ZIP) [file pone.0298601.s001.zip › Data/地级市Z_F工作报告.zip!/░▓╗╒/░▓╟∞╩╨/└┤╫╘╣1⁄2╓┌║┼í╛┬φ┐╦╩2╛▌═°í┐_2013.docx]

**政 府 工 作 报 告**

**——2013年1月7日在安庆市第十六届人民代表大会第一次会议上**

市长  虞爱华

各位代表：

现在，我代表市人民政府，向大会报告政府工作，请予审议，并请列席会议的市政协委员和其他同志提出意见。

一、过去四年的工作回顾

市十五届人大一次会议以来，本届政府在市委的坚强领导下，认真贯彻落实科学发展观，以全面转型、加速崛起、富民强市为主线，着力推进实力安庆、活力安庆、文化安庆、和谐安庆建设，圆满完成“十一五”目标任务，顺利实施“十二五”规划，经济社会发展取得令人鼓舞的崭新成就。

**——过去四年，经济持续快速发展。**预计2012年全市地区生产总值1300亿元以上,跨越6个百亿台阶，总量居全省第3位；财政收入170.3亿元，跨越10个十亿台阶，总量居全省第4位。5个县（市）经济总量过百亿，3个县（市）财政收入过十亿。**工业化进程不断加快。**工业经济比重超过50%。石油化工、装备制造、纺织服装等支柱产业快速发展，产值过千亿元，是2008年的1.8倍。规模以上工业企业发展到1348家。国际纺织工业城、汽车零部件基地建设扎实推进。建立5亿元战略性新兴产业发展引导资金，高新技术产业增加值增长2.3倍。非晶软磁生产设备研发填补国内空白。关停并转高能耗、高污染企业189家，节能减排任务如期完成。**农业稳步提升。**粮食连年增产，去年总产超过270万吨。新建城郊蔬菜基地2.39万亩。畜禽规模养殖比重提高23个百分点。实施农业产业化转型倍增计划，新增省级龙头企业52家、国家级3家，龙头企业户数居全省之首。农村资金互助试点经验全省推广。农民技能和职业农民培训50万人次。耕地规模经营面积135.5万亩，居全省前列。新增农民专业合作社2408家。新增国家和省级名牌农产品58个。政策性农业保险受益187万户次。**服务业全面发展。**服务业增加值是2008年的1.7倍。“万村千乡”市场工程、“新网工程”走在全省前列，家电和汽车摩托车“两下乡两换新”销售额全省领先。迎江区成为全省城市商贸服务业发展示范区。国家级再生资源回收利用基地一期工程建成运营。粮食物流园等加快建设。社会消费品零售总额为2008年的两倍。新增国家4A景区9个，天柱山荣获国家5A景区和世界地质公园称号，五千年文博园等3家企业成为国家文化产业示范基地。旅游业总收入达248亿元。银行业金融机构达26家，存款余额近1700亿元，总量和增量分别居全省第3位和第2位；贷款余额达870亿元，总量和增量分别居全省第4位和第3位。

**——过去四年，城乡面貌显著变化。基础设施建设加强。**新改建干线公路721公里。六潜高速建成通车，宁安城际铁路及安庆长江铁路大桥、望东长江公路大桥、岳武高速等重大基础设施项目扎实推进。新增5000吨级泊位5个，安庆港实现对台直航。石门湖航道疏浚工程开工建设。实施天柱山机场升级改造，开通北京、上海、广州等5条航线。花凉亭等288座大中小型水库除险加固完成，大沙河流域治理完工。500千伏文都和双岭变电站建成投运。改造提升邮政网点111处，宽带数据、广播电视网络覆盖90%以上行政村。**城镇建设加快。**新一轮城市总体规划获批实施，“双百”城市框架基本形成。主城区新改建道路31条，整治街巷40条。市区供水、用气普及率均超过90%，污水处理率86.2%。开展小城镇建设“宜城杯”竞赛，推进县城和中心镇建设。在全省率先启动国家森林城市创建工作，实施七大绿化工程，植树100万株。获中国十佳绿色城市称号。**新农村建设加速。**新建“村村通”公路6026公里。解决213万农村人口饮水安全问题。实施新农村建设示范项目1152个，建成示范点390个。完成危房改造37728户，争取资金占全省三分之一。潜山县村庄整治工作走在全省前列。治理水土流失面积500平方公里。耕地总量保持动态平衡。造林70.5万亩，森林覆盖率提高到36.2%。

**——过去四年，改革开放不断深入。**国有集体企业改革和事业单位转企改制任务基本完成。新增私营企业10570户，非公经济比重达58.2%，提高5.8个百分点。农村综合改革和集体林权制度改革不断深入。扩权强县和扩权强镇试点稳步推进，宿松被列为“省直管县”试点。启动实施营业税改征增值税。推进市直行政事业单位国有资产管理改革。国库集中支付、政府采购改革进一步完善，市直公务支付卡全覆盖。政府投资项目审计监督工作进一步加强。8家农村信用社改制组建农村商业（合作）银行，小额贷款公司发展到44家、融资性担保公司43家。安徽盛运机械股份有限公司成功上市，华茂纺织股份公司发行债券11.4亿元。文艺院团转企改制全面完成，文化体制改革连续两年荣获全国先进，再芬黄梅艺术股份公司获全国先进单位称号。不断深化基层医药卫生体制改革，县级以下医院全面实行药品零差率销售。市开发区进入国家级行列，省级以上开发园区达18家，居全省首位。着力承接产业转移，引进亿元以上项目398个、市外资金2500多亿元，利用外资9.1亿美元。外贸进出口总额达11.8亿美元，是2008年的2.1倍。新增高新技术企业34家、高新技术产品61项。现有中国名牌6个、中国驰名商标13件、安徽名牌99个，名牌总数全省第二。

**——过去四年，人民生活明显改善。**民生工程累计投入财政资金182.3亿元。实施城乡居民收入倍增规划，城镇居民人均可支配收入年均增长13.3%，农民人均纯收入年均增长13.9%。养老保险制度实现全覆盖，参加率91.1%。医保基金支付比例居全省前列。工伤保险参保人数达31.4万。城镇新增就业22万人，下岗失业人员再就业10万人，城镇登记失业率控制在4.2%以内。建立社会救助标准与物价上涨联动机制。市区低保标准由每人每月245元提高到360元，农村低保标准由每人每年860元提高到2100元。宜秀区五保对象集中供养率达92.8%，在全省领先。新建光荣院3所。向12440位80岁以上老人发放高龄补贴。大力实施整村推进工程，脱贫40万人，扶贫开发工作全国先进。少数民族和民族聚居地区加快发展。住房公积金制度覆盖面进一步扩大，向5.37万户家庭发放贷款53.1亿元。新建保障性住房80475套，对18496户发放廉租住房补贴，实物配租保障9162户。

**——过去四年，社会事业全面发展。**“两基”教育扎实推进，桐城市获全国先进单位称号。义务教育经费保障机制进一步完善，基础教育优势地位不断巩固。职业教育和高等教育加快发展，桐城师专、黄梅戏艺术职业学院先后获批，安庆技师学院成功组建。投入资金17.41亿元，加固改造校舍275.26万平方米。改扩建公办幼儿园97所。新农合参加率98.8％，受益人数达593.8万人次。医疗废弃物实现集中统一处置。市县两级10个疾病预防控制中心、10个医疗救治项目投入使用，医疗卫生服务体系建设得到加强。全国听力语言康复事业规范化建设试点取得实效。建成黄梅戏艺术中心和中国黄梅戏博物馆，独秀园等公益文化设施免费开放。建成乡镇综合文化站152个、广播电视村村通工程9606个、农家书屋1642个、公共电子阅览室101个。完成第三次全国文物普查任务。“安庆大讲坛”等文化活动品牌效应显现。成功举办第五、六届中国黄梅戏艺术节，荣获全国节庆品牌50强，被省政府命名为黄梅戏传承创新基地。健全人口计生服务网络，完善计生奖扶政策，人口自然增长率控制在6‰以内。完成第六次全国人口普查任务，企业“一套表”制度顺利实施。成功举办第十二届市运会。安全生产形势持续好转。应急管理体系建设得到加强。食品药品放心工程深入实施。平安安庆建设力度加大，普法教育有序推进，公安“三项建设”和社会管理综合治理取得实效。全面开展争创全国双拥模范城活动，岳西荣获全国双拥模范县称号。成功创建省级文明城市。残疾人、老年人和妇女儿童权益得到保护，国防动员、人民防空、国家安全、物价、地震、气象、档案、地方志、民族、宗教、外事、侨务、对台、红十字会等工作取得新的成绩。

刚刚过去的2012年，全市上下紧扣主题主线，坚持“稳中求进、好中求快”总基调，有效化解突出矛盾和问题，千方百计保企业、稳增长、调结构、转方式、惠民生，保持经济社会平稳较快发展势头。预计地区生产总值、社会消费品零售总额、固定资产投资、城乡居民收入等主要经济指标基本完成年度目标任务。财政收入增长8.6%。一年来，我们突出抓了以下五个方面工作：**一是扶持实体经济。**实施促进经济平稳较快发展25条措施，为112户企业争取扶持资金5600万元，向694户困难企业发放就业培训和稳岗补贴1964万元，减征企业社保费1.5亿元。财政安排外贸进出口激励资金2000万元。拨付战略性新兴产业扶持资金1.04亿元，飞凯高分子、西锐重工、金张科技等企业快速成长。发明专利授权55件。全年社会融资总量250亿元，增长31.5%。完成工业投资570亿元，增长20%。新增规模以上工业企业203家。**二是抓好项目建设。**实施“861”和“3231”项目483个，完成投资350亿元。石化炼化一体化、华泰林浆纸一期、华港风力发电、旺旺四期等项目建成。中船马克柴油机、曙光煤制氢和丁辛醇、华茂佰斯特高档面料、高铁动车配件、桐城万信光电科技、安踏泰亚鞋业等项目加快建设，下浒山水库、神华安庆电厂二期两台100万千瓦机组、华谊化工新材料等项目前期工作取得突破。**三是做强承接平台。**市开发区加快扩容升级，高新产业园、铸锻产业园建设加快。化工新材料产业集中区获省政府批准，凤凰片骨干路网基本建成，六大公用工程全面启动。国家石化质检中心建成使用，国家高分子材料质检中心建设进展顺利。市科技创业辅导中心获国家级示范服务平台称号。省级开发区竞相发展，全市园区工业产值达1000亿元，增长40%。**四是统筹城乡发展。**油料、棉花、茶叶、肉类、禽蛋及水产品持续增产。桐城羽绒、望江棉纺产业板块获批省级农业产业化示范区。农产品加工值超过700亿元，居全省第二。全面推进美好乡村建设，32个乡镇实施清洁工程，整治村庄334个，改造危房29889户，农村生产生活环境进一步改善。推动大别山集中连片贫困地区扶贫开发。42项民生工程全面完成，财政民生支出比重达80%。居民消费价格指数上涨2.2%，低于全省全国水平。城镇化率达45.9%，比上年提高两个百分点。**五是加快中心城市建设。**与中建七局、八局、中城建集团、中坤集团战略合作，借力加快重大基础设施项目建设。顺安路三期、长风路等8个项目完工。人民路综合改造、康熙河（新河）水系治理、花亭大沟整治、一水厂迁建等18个城建重点工程扎实推进。市政务中心建设加快，市体育中心、八县（市）体育场馆建设全面启动。客运中心站投入运行。市区开工建设还建房380.5万平方米，建成227.8万平方米。推进城区环卫作业方式改革，市容环境明显改观。

四年来，在推进经济社会发展的同时，我们高度重视政府自身建设，自觉接受市人大及其常委会的法律监督和市政协的民主监督。办结市人大代表议案建议572件、市政协提案1831件，办结率均达100%。清理规范性文件187件，废止、修改81件，办理行政复议案件427件。开通政务信息公开网站1066个，政务公开工作全省领先。持续开展“主要领导述效能”、“百名科长群众评”、“千项办件看效能”活动。清理规范行政审批，取消或下放126项。推进“两集中、两到位”，实行并联审批、全程代理和“绿色通道”制度，进一步提速增效。完善网络问政平台，网民留言办结率超过97%。深入开展领导干部大走访、带案下访、开门接访活动，在全省率先设立涉法涉诉联合接访服务中心，集中化解信访积案800多件。全面开展廉政风险防控，加强招标采购监管，开展工程建设等领域专项整治，查处违法违纪案件582件。

各位代表、各位委员、各位同志，四年的发展有目共睹、令人振奋，四年的成就来之不易、可圈可点。这是省委、省政府和市委坚强领导的结果，是市人大和市政协监督支持的结果，是全市人民共同奋斗的结果。在此，我代表市人民政府，向全市广大工人、农民、知识分子、干部、驻宜解放军指战员、武警消防官兵和政法干警，向各民主党派、各人民团体和社会各界人士，向关心支持安庆现代化建设的中央和省驻宜单位、海内外友好人士，向在我市创业的投资者、建设者，致以崇高的敬意，表示衷心的感谢！

在充分肯定成绩的同时，我们也要清醒地认识到，我市发展不足、发展不优的问题仍然突出，转型发展的任务艰巨繁重，中心城市辐射带动能力还要进一步加强，县域经济发展还要进一步加快，居民收入、公共服务和社会保障水平有待进一步提高，少数部门服务发展的意识和水平有待进一步提升，少数地方自信心还要进一步增强。对此，我们一定认真加以解决。

二、未来五年的发展目标和主要任务

今后五年，**必须全面贯彻落实党的十八大精神，按照市第十次党代会要求，以科学发展为主题，以加快转变经济发展方式为主线，强力推进新型工业化、信息化、城镇化和农业现代化，建设重要的石油化工、装备制造、纺织服装、文化旅游产业基地，打造宜居宜业宜游的区域性中心城市，不断向经济强市、文化强市和生态强市迈进。**

经过五年的努力，实现经济总量保持全省前列，指标增幅高于全省，居民收入达到全省平均水平，确保完成节能减排任务，为全面建成小康社会打下坚实基础。

**——中心城市建设要有新突破。**按照“城市建设是重要的民生工程”的理念，加快“大宜城”建设。**加强路网建设，让城市更加通畅。**以拉开框架为重点，拓宽进出口道路。加快建设中心城市至周边县城快速通道，建成外环道路。**加快升级改造，让老城更加靓丽。**坚持“控制一片、放开一线”，用3年左右时间完成老城区背街后巷、老旧小区、农贸市场改造。加大城市地下管网建设力度。推进退建还绿，新建一批街头小游园。深化文明创建，美化人居环境，让市民充分享受城市发展之利。**完善市政功能，让新城更具人气。**增强教育、卫生、文化、体育、商务、金融、会展等城市功能。完成康熙河（新河）水系景观带建设，创建国家5A景区。加快白泽组团规划建设。加大还建房建设力度，力争用两年左右时间还清“旧账”、不欠“新账”，逐步实现先安置后征迁。**加大统筹力度，让县城更具活力。**支持桐城深度融入合肥经济圈，宿松建设三省交界新兴工贸城市，推进怀宁、枞阳与中心城区同城化发展，推动潜山建设新型旅游休闲城市，力争早日“撤县建市”，望江打造滨江产业新城，岳西和太湖依托特质资源打造生态旅游城市。加快县域经济发展，奋力在全省科学发展中争先进位。

**——综合交通建设要有新进展。**坚持交通先行，完善交通投融资和建设管理机制，建设东进西出、南通北达的立体综合交通体系。**打造重要铁路节点城市。**建成宁安城际铁路（安庆段），力争开工建设阜阳至景德镇铁路（安庆段）、合安城际铁路（安庆段）、合九铁路（安庆段）电气化改造、安庆至天柱山轻轨等，基本形成“三横两纵”跨区域铁路快速干线通道。**巩固公路交通枢纽地位。**建成济广高速（安庆段）、岳武高速（安庆段）、济祁高速（安庆段），建设北沿江高速（安庆段），形成“四纵四横”高速公路网。改造、升级、加密境内国省道干线公路，重点推进一级公路建设。**加快长江岸线资源开发。**加强安庆、枞阳、望江、宿松四大港区建设，打造铁水联运枢纽，垦地合作建设现代物流园区，港口年吞吐能力超过4000万吨。**加强安庆空港建设。**完善天柱山机场通航条件，力争开工建设新机场。

**——开发区建设要有新提升。**坚持扩容升级与提质增效并举，建成两大千亿园区，打造全市经济新的增长极。**推进资源整合。**本着共建双赢的原则，适时重组宜城板块园区，逐步形成市开发区“一区多园”，化工新材料产业集中区凤凰、山口、皖河农场“三片联动”的发展格局。**坚持集约发展。**突出首位产业，促进产业集群发展，鼓励工业企业退城进园。支持市开发区依托高等院校和科研院所，建设高科技产业园和高校产业园。鼓励创建省级以上技术中心、工程中心、博士后工作站、院士工作站。推进化工新材料产业集中区与央企深化合作，打造经济爆发式增长点。着力提高园区投资强度、产出率标准。配套发展生产性服务业。**创新体制机制。**推行园区管理企业化、资源资本化、投资多元化，不断增强发展活力和动力。推动省级园区错位发展、差异化竞争，支持桐城开发区争创国家级。激励企业上市，力争每个县（市、区）新增1家以上上市公司。

**——美好乡村建设要有新形象。**顺应农民过上美好生活的新期待，统筹推进城乡发展，突出规划引领，打造10条美好乡村示范带，建设生态宜居、兴业富民、文明和谐的新农村。**实施村庄建设工程。**整体推进水、电、路、气等基础设施建设，优化房舍布局，打造一批布局合理、特色鲜明、设施配套、环境整洁的中心村。**实施环境整治工程。**建立农村保洁制度。推进水库灌区、蓄滞洪区、长江河段整治工程，加强农田水利建设，改善农业生产条件。**实施兴业富民工程。**加快建设现代农业示范区，做大做强龙头企业、专业合作社和专业大户，发展农产品精深加工，建设旅游特色乡镇，促进产村相融。大力推进大别山集中连片贫困地区扶贫开发，改善基础设施，强化特色产业支撑，加快脱贫致富步伐。**实施土地整治工程。**加强基本农田建设，增强农业综合生产能力。探索土地出让收益城乡统筹使用机制，提高农村土地利用率。**实施乡风文明工程。**深化农村精神文明创建，弘扬乡村文明新风，培育一批小康文明村。实施文化惠民工程，丰富农民精神文化生活。**实施管理创新工程。**强化基层组织建设，推行“阳光村务”。建设农村社区综合服务中心，实行一站式服务。健全农村居民保障体系，创新留守人员关爱服务机制。

**——文化建设要有新发展。**弘扬“崇文尚德、务实创新”安庆精神，深化群众性精神文明创建活动，把文化底蕴转化为精神动力，把文化资源转变为产业优势。**着力打造“有戏”的城市。**充分运用多种手段，吸引和培育黄梅戏新生代、新戏迷，送戏进企业、进社区、进学校、进机关，建设黄梅戏大观园，提升影响力，扩大覆盖面，延伸产业链。不断推出精品力作，努力培养名角新人，争创全国“五个一工程”奖。**着力培育“有形”的产业**。积极参与皖南国际文化旅游示范区建设，推进旅游业由公务接待型向产业发展型转变，由旅游观光型向休闲度假型转变。重点发展文化旅游、创意传媒等文化产业，建成天柱山、浮山旅游度假区等一批重点项目，改造提升倒扒狮街、孔城老街等历史文化街区，挖掘胡玉美等“中华老字号”文化价值，让安庆文化有“说头”、有“看头”，更有“赚头”**。着力传承“有名”的文化。**加强古皖文化、戏曲文化、桐城派文化和禅宗文化的研究开发。保护振风塔等历史遗存，修复大观亭等历史遗址，建成薛家岗遗址公园等标志性文化设施，实现文化惠民。

**——社会建设要有新成效。**更加重视改善民生，推进管理创新，建设和谐社会。**努力办人民满意教育。**实施学前教育公共服务体系覆盖工程，确保城乡学龄前儿童入园率达90%以上。调整优化学校布局，推进义务教育学校标准化建设，推动高中教育优质特色多样化发展。提升职业教育和高等教育内涵，支持安庆师范学院创建综合性大学。促进民办教育健康发展。**实现更高质量就业。**广泛开展就业培训，力争新增劳动力全部接受岗前技能培训。落实创业扶持政策，以更好的创业带动更多的就业。健全政府购买服务机制，为就业困难群体提供更多的公益性岗位。**健全城乡保障体系。**以提标扩面为重点，推进城乡社会保险制度全覆盖。积极发展居家养老服务，壮大老龄服务事业和产业。加快推进住房保障体系建设，扩大住房保障受益面。**提高群众健康水平。**加强医疗资源整合，健全公共卫生服务体系，继续深化医药卫生体制改革。广泛开展全民健身活动，增强全民身体素质。坚持计划生育基本国策，促进人口长期均衡发展。**加强社会管理创新。**改进政府提供服务方式，健全网格化、信息化、多元化、专业化的服务管理体系。建立重大决策社会稳定风险评估机制，完善公共突发事件应急管理体系。创新流动人口和特殊人群管理服务。

**——生态文明建设要有新举措。**下决心创建国家环保模范城，进一步改善人居环境和重化工城市形象。**开展污染减排专项行动。**深化大气污染排放治理，加强污水处理能力建设，实行危化废弃物集中处置,确保空气质量优良率、城市污水集中处理率和工业固废综合处置利用率均达到90%左右。**开展节能降耗专项行动。**加快淘汰落后生产能力，坚决控制新上高能耗、高污染项目。完善能评机制，支持节能改造，加强能效监管，逐步实现单位GDP能耗低于全国平均水平。**开展环境保护专项行动。**落实安庆石化1300米安全卫生防护距离要求，退建腾地，植树种草。坚持“绿色化工、循环经济、生态园区”的理念，完善化工新材料产业集中区规划，着力建设花园式示范园区。坚持“上大压小”，推进集聚集群发展，强力关停并转一批中小化工企业。加强生态功能区、自然保护区建设，推进小流域综合治理，减少农业面源污染。实施百万亩森林增长提质工程。严格耕地保护和水资源管理，加强矿产资源有序开发、合理利用，建设山更绿、水更净、天更蓝的美好家园。

**三、2013年重点工作**

今年是全面贯彻党的十八大精神的第一年，是新一届政府的开局之年。做好今年各项工作，对完成“十二五”规划和今后五年目标至关重要。必须认真贯彻落实中央和省委、省政府决策部署，加快转型升级，加速跨越崛起，奋力开创经济社会更好更快发展的新局面。

综合考虑，今年全市经济社会发展的主要预期目标是：地区生产总值增长11%，财政收入增长10%，社会消费品零售总额增长15%，固定资产投资增长20%，城镇居民人均可支配收入增长13.5%，农民人均纯收入增长14%，城镇登记失业率控制在4.2%以内，人口自然增长率控制在6‰以内。确保完成节能减排任务。

**（一）着力推进新型工业化。加快工业转型升级。**加强三大千亿产业建设，大力发展化工新材料，提升装备制造技术水平，延伸纺织服装产业链。充分发挥战略性新兴产业引导资金作用，重点支持新材料、节能环保、高端装备制造等产业发展。优化非公经济发展环境，进一步清费减税，促进中小企业加快发展。实施工业经济“双百”工程，持续扶持100家招商引资企业和100家本土企业，促进其做大做强。**加强重点项目建设。**实施“861”和“3231”项目440个，完成投资360亿元。推进石化炼化一体化、中船马克柴油机、泰发能源等项目投产见效，建成卫康标准化药品生产基地，开工建设华谊化工新材料、神华安庆电厂二期、泰达合成树脂、中明环保材料等项目,加快推进华茂国际纺织工业城、安踏工业园、曙光化工园等项目建设，积极争取桐城抽水蓄能电站项目。**提升招商引资实效。**组建8个重点产业招商组，推进重点区域招商，加强境外招商。大力引进科技含量高、有核心技术、成长性强的企业落户。深化安庆籍商界精英报效桑梓活动。力争引进亿元以上项目100个、10亿元以上项目20个。**增强开发区功能。**完善市开发区三期路网，推进中兴大道以西片区整体开发，建成高新技术中小企业孵化器，启动建设高校产业园区、公共保税仓库。健全化工新材料产业集中区凤凰片路网，加快工业用水、热电、科技中心等工程进度。全面启动省级园区多层标准化厂房建设。**狠抓节能减排工作。**落实石油化工、建材等重点行业节能降耗措施。严格监管化工企业，淘汰工艺落后企业，坚决关停排放不达标企业。加强城镇污水处理能力建设，建成城区3、4号污水泵站，推动北部新城污水处理厂、城东污水处理厂二期建设，提高污水处理率。推进建筑、交通运输、商业和公共机构等领域节能工作。

**（二）着力加强城乡统筹。提升“三农”工作水平。**扶持农业产业化龙头企业做大做强，力争新增国家级龙头企业1家、省级30家以上。支持纺织服装、食品加工等农业特色板块经济发展。推进耕地适度规模经营，岳西菖蒲、大观海口、怀宁平山、宿松洲头争创省级现代农业示范区，支持桐城范岗创建国家级示范区。启动省级蔬菜、家禽、水产品交易中心建设。新增农民专业合作社520家，技能培训6万人。加大城郊商品蔬菜基地建设力度。进一步拓展政策性农业保险覆盖面。**加强农田水利建设。**力争开工建设下浒山水库。启动皖河治理工程。继续抓好小农水重点县建设，完成62座病险小型水库除险加固、16条中小河流整治，治理水土流失168平方公里。解决33万农村人口饮水安全问题。大力实施土地增减挂项目，建成高标准基本农田38.37万亩。**推进美好乡村建设。**编制美好乡村发展规划，重点抓好207个中心村建设、1000个自然村治理。实施40个乡镇农村清洁工程，完成3万户危房改造，力争通过几年努力，在全省率先完成农村危房改造任务。改造升级县乡公路200公里，延伸通村公路310公里，加固改造危桥151座。加快大别山集中连片贫困地区扶贫开发，实施150个“整村推进”工程。深化对口扶持，推进少数民族和民族聚居地区“共同发展”提升行动。完成20万亩森林增长提质任务。

**（三）着力提升服务业。**以人民路改造为契机，加大老城改造力度，打造休闲、购物、娱乐为一体的历史文化景区。建成迎江世纪城商贸综合体、华茂和汇峰广场综合体主体工程，加速推进皖江农产品生产物流中心、五里庙集装箱综合物流园、再生资源回收利用基地二期等项目建设。开工建设八佰伴商业综合体。启动光彩大市场改造升级工程。建设城区机械化生猪屠宰场，改建5个标准化农贸市场。启动黄梅戏大观园建设。面向全国征集黄梅戏优秀剧本，开展年度最具人气黄梅戏演员评选，启动合肥等地“黄梅戏大舞台”连锁演艺场馆建设，促进黄梅戏事业大繁荣、产业大发展。建成桐城文化博物馆，加快建设岳西国际养生文化产业园、太湖五千年文博园二期、天柱山地质博物馆。加快发展乡村旅游，建成8家四星级以上农家乐。创建国家4A景区2个以上。加强旅游宣传推介，力争游客达到3500万人次以上，旅游总收入280亿元以上。启动安庆金融集聚区建设，吸引股份制商业银行来宜设立分支机构，支持农村商业（合作）银行、村镇银行发展。大力推进企业上市。启动实施“债务融资倍增计划”，落实城投公司保障房债券、市开发区建投公司企业债发行。加强担保公司和小额贷款公司监管。鼓励金融机构增加信贷投放，确保信贷增长高于全省平均水平。

**（四）着力加快新型城镇化。加强城镇规划和建设。**重点实施中心城市建设“十大工程”，即市政务中心，市体育中心和全民健身中心综合训练馆，东部新城区综合医院一期，人民路综合改造，湖心路等4条道路黑色化，菱湖风景区水环境综合治理，外环北路一期、独秀大道三期和勇进路二期建设，康熙河（新河）整治，城区出入口通道拓宽，荣升街、天后宫街等10条背街后巷改造升级。同时，启动高速安庆出入口迁址规划建设，建成环城南路二期，推进环城东路建设。建成潜江路、柘山路、祥和路，开工建设沿江东路中段、秦潭路、天柱山西路、文苑路南段，实施中兴大道铁路平交口改造，启动安庆火车站站房和站前广场建设。加大城市公交投入，增加公交线路，延长运行时间，建设秦潭湖、红水塘公交枢纽站。标本兼治，分期实施，全面推进花亭大沟整治、德宽路地质灾害点治理。清理占道经营，畅通“微循环”。推进25个老旧小区和“城中村”改造。启动白泽组团土地一级整理。创建省级森林城市两个、森林城镇25个，确保通过国家森林城市创建验收。深化“宜城杯”竞赛活动，推动每个县（市、区）建设两个风情小镇。**强化城镇管理。**严格执行规划，全面清理城市规划区范围内用地，推进低效利用土地二次开发。严禁违法用地，大力拆除违法建筑，打击各种干扰施工的违法行为，优化城镇建设环境。规范户外广告管理。规范摊群点，整治占道经营、车辆乱停乱放，加强建筑渣土运输管理，全面落实门前三包，深化文明城镇创建。**完善城际交通。**加快建设宁安城际铁路、望东长江公路大桥及接线、岳武高速、石门湖航道整治、长风港等项目。积极协助开工建设枞阳至池州长江公路大桥，加快推进北沿江一级公路宿松段、潜山野寨至桃花铺、枞阳至圣埠、桐城至大龙山、大龙山至望江等国省道干线改扩建，启动安庆至罗岭、怀宁至枞阳快速通道规划建设。

**（五）着力改善人民生活。提高民生工程质量。**集中力量实施省定33项民生工程，财政投入58亿元。健全项目管护机制，实现民生工程效益最大化。投资36.23亿元，开工建设保障性住房26366套，基本建成15700套。深入实施居民收入倍增规划，确保城乡居民收入较快增长。保持物价基本稳定，居民消费价格涨幅控制在3.5%左右。**加强就业和社会保障工作。**重点做好高校毕业生和被征地农民就业工作，大力促进农村劳动力就近就地就业。推行医疗保险异地结算。提高城乡低保、重度残疾人生活救助标准，健全临时救助制度，推进城乡医疗救助一站式服务。完善新型农村合作医疗制度,确保参加率稳定在97％以上。加强对生活无着落人员社会救助，广泛开展关爱留守人员活动，推行城乡孤儿救助和集中供养，免除特殊教育学生的生活费。提高“五保”集中供养率，建设30家城市社区养老服务中心（站）、20家示范性农村敬老院，扩大社会化养老覆盖面。**加快教育文化卫生等事业发展。**强力推进市区教育布局调整，适度增加农村偏远地区小学教学点。大力发展学前教育，完成82所乡镇公办幼儿园建设。启动桐城师专新校区建设。扎实开展文化下基层活动，推进市博物馆、图书馆、文化馆、美术馆建设。深化县级公立医院改革，完善基层卫生服务网络。广泛开展全民健身运动，全面推进省运会及残运会筹备工作。深化人口服务管理改革创新，综合治理出生人口性别比，提高出生人口素质。完成第三次全国经济普查任务。推进残疾人社会保障和服务体系建设试点。深化殡葬改革，规划建设城市新的公益墓园。做好科普、档案、地方志、外事、侨务、老龄、红十字会等工作。

**（六）着力创新社会管理。以群众工作统揽信访工作。**引导群众依法有序表达诉求，坚持领导干部开门接访和积案化解，切实解决群众反映强烈的问题。加强调解工作，依法果断处置“两堵两闹”等违法行为。积极开展“六五”法制宣传教育、法律援助和社区矫正工作。着力构建和谐劳动关系，确保农民工工资不拖欠。**健全社会事务管理机制。**推进社区综合管理体制改革，增强社区自治和服务功能，加大社区事务财务公开力度。强化企事业单位、人民团体社会管理和服务职责，引导社会组织健康有序发展，发挥群众参与社会管理的基础作用。加快推进流动人口基本公共服务均等化。重视民族工作，加强宗教事务管理。充分支持工会、共青团、妇联等人民团体发挥重要作用。**健全公共安全管理机制。**深入开展平安安庆建设，强化社会管理综合治理，依法防范和惩治违法犯罪活动，保障人民生命财产安全。严格落实安全生产责任，加强重点行业、重点领域和重点部位的安全监管，深入开展隐患排查治理，彻底关闭小煤矿和烟花爆竹生产企业，杜绝重特大安全生产事故发生。切实加强学校及周边治安环境整治，保障校园安全、校车安全。强化食品药品安全日常监管和专项整治。健全应急管理体系，切实抓好地震、地质、气象等灾害防治。支持驻宜部队建设。加强国防教育、国防动员、人民防空和民兵预备役工作，深入开展“双拥”共建活动。

四、切实加强政府自身建设

新一届政府使命光荣，责任重大。需要我们甘于奉献，勇于担当，善于落实。必须以开明开放的理念、创新创造的精神、敢抓敢管的作风，埋头苦干，取信于民，造福百姓，努力建设为民务实清廉政府。在实际工作，坚决做到“四政”：

**一是全心全意为民理政。**始终把人民放在心中最高位置，问政问需问计于民，了解群众所思所盼所急，到基层掌握实情，到一线化解矛盾，到现场推进工作。感情上与群众“心连心”、工作中与群众“面对面”、解决问题“实打实”，多谋民生之利，多解民生之忧。深化政府机构和行政审批制度改革，健全网上审批和电子监察系统。完善市区财政体制，充分调动各区工作积极性。改善公共服务，优化办事流程，让群众一看就明白、一听就清楚、一次就办好，切实解决办事难问题。

**二是不折不扣依法行政。**运用法治思维和法治方式管理经济社会事务，坚持依法依规、公开公正，重大事项决策前必须经过法律论证、社会听证和舆情评估。自觉接受人大及其常委会的法律监督、政协的民主监督、媒体的舆论监督和社会各界的群众监督，认真办好人大议案、代表建议和政协提案，定期向人大、政协报告、通报政府工作。完善政府工作规则，深入推进政务公开，促进决策程序依法规范、过程科学民主、结果经得起检验。健全行政执法责任制，落实责任追究制，创造优良的发展环境。

**三是立言立行高效施政。**坚持雷厉风行、说干就干，一抓到底、干就干好，只为成功找办法，不为困难找借口。注重实际和实效，开短会、发短文、讲短话。推行政府工作项目化，项目工作责任化，工作责任具体化。持续深化效能建设，坚决防止“在岗不在干、在干不在行”等倾向，着力整治“不作为、慢作为、乱作为”等问题，努力提高公务员队伍整体素质和服务水平。对事关全局的大事、改善民生的实事、久拖不决的难事，加强督查督办，严格问责问效。

**四是慎独慎微廉洁从政。**严格执行党风廉政建设各项规定，坚决按照中央和省委要求，切实改进工作作风，密切联系群众。严禁一切不必要的检查评比表彰活动，严禁收受礼品礼金，严禁铺张浪费。扎实推进惩治和预防腐败体系建设。强化权力制约和监督，用制度管权管事管人。加强政府重点投资项目、重大资金支出、重要公益性基金监管和跟踪审计，加强行政事业单位公有资产管理，强化重要领域、关键环节行政监察，严肃查处违法违纪案件。切实履行“一岗双责”，始终保持“做人有底气、干事有锐气、为官有正气”，做到干部清正、政府清廉、政治清明。

各位代表、各位委员、各位同志，新的征程已经开启，新的形势催人奋进。经验表明，一个地方的发展，只有真干才能真上，只有大干才能大上，只有实干才能有实绩。我们将始终“把人民的期待放在心上，把安庆的发展扛在肩上”，深入贯彻落实党的十八大精神，在中共安庆市委的坚强领导下，绝不空谈、绝不懈怠、绝不敷衍、绝不自满，为建设美好安庆而努力奋斗！
